# Investigations into the Toxicology of Spirolides, a Group of Marine Phycotoxins

**DOI:** 10.3390/toxins4010001

**Published:** 2011-12-30

**Authors:** Rex Munday, Michael A. Quilliam, Patricia LeBlanc, Nancy Lewis, Pamela Gallant, Sandra A. Sperker, H. Stephen Ewart, Shawna L. MacKinnon

**Affiliations:** 1 AgResearch, Ruakura Research Centre, Private Bag, Hamilton 3123, New Zealand; Email: rex.munday@agresearch.co.nz; 2 National Research Council of Canada, Institute for Marine Biosciences, 1411 Oxford Street Halifax, Nova Scotia B3H 3Z1, Canada; Email: michael.quilliam@nrc.gc.ca (M.A.Q.); patricia.leblanc@nrc.gc.ca (P.L.); nancy.lewis@nrc.gc.ca (N.L.); pamela.gallant@nrc.gc.ca (P.G.); sandra.sperker@nrc.gc.ca (S.A.S.); stephen.ewart@novaceutics.ca (H.S.E.)

**Keywords:** spirolides, marine phycotoxin, acute toxicity, *Alexandrium ostenfeldii*, seafood poisoning

## Abstract

Spirolides are marine phycotoxins produced by the dinoflagellates *Alexandrium ostenfeldii* and *A. peruvianum.* Here we report that 13-desmethyl spirolide C shows little cytotoxicity when incubated with various cultured mammalian cell lines. When administered to mice by intraperitoneal (ip) injection, however, this substance was highly toxic, with an LD_50_ value of 6.9 µg/kg body weight (BW), showing that such *in vitro* cytotoxicity tests are not appropriate for predicting the *in vivo* toxicity of this toxin. Four other spirolides, A, B, C, and 20-methyl spirolide G, were also toxic to mice by ip injection, with LD_50_ values of 37, 99, 8.0 and 8.0 µg/kg BW respectively. However, the acute toxicities of these compounds were lower by at least an order of magnitude when administration by gavage and their toxic effects were further diminished when administered with food. These results have implications for future studies of the toxicology of these marine toxins and the risk assessment of human exposure.

## 1. Introduction

The class of macrocyclic imines known as spirolides was first identified in toxic extracts of the digestive glands of mussels and scallops from the Atlantic coast of Nova Scotia, Canada, in the early 1990s [1]. The distinguishing feature of these compounds is the presence of a cyclic imine moiety, which has been found elsewhere only in the marine toxins known as pinnatoxins, pteriatoxins, prorocentrolides, spiro-prorocentrimine, and gymnodimine [2,3,4,5]. The marine dinoflagellate *Alexandrium ostenfeldii* (Paulsen) Balech & Tangen was identified as the source of spirolides in Nova Scotia [6,7]. This finding was surprising because *A. ostenfeldii* had been previously reported as a source of the neurotoxins associated with paralytic shellfish poisoning (PSP), an unrelated toxic syndrome [8]. 

The contamination of shellfish with spirolides is a food safety and human health concern. The unusual cyclic imine feature is thought to be the pharmacophore responsible for the toxic effects of these substances in experimental animals [9]. Spirolides are described as “fast-acting” phycotoxins, because of the rapidity of the onset of neurological symptoms and short time to death [10]. The molecular basis for these effects may, at least in part, be due to the recently reported antagonist actions of spirolides at nicotinic-type and muscarinic-type acetylcholine receptors [11,12]. 

In order to better understand their toxicity, we have studied the effects of 13-desmethyl-spirolide C (**13dmC**) on mammalian cell lines and compared the LD_50_ of various spirolides in mice, via different routes of administration. 

## 2. Materials and Methods

Trifluoroacetic acid (TFA) and HPLC-grade acetonitrile, methanol, and dichloromethane were purchased from Caledon (Georgetown, ON, Canada). Ethanol (95%) was purchased from BDH (Poole, UK) and Tween-60 from Merck (Darmstadt, Germany). Sephadex LH-20, glutamine, nicotine and insulin were purchased from Sigma-Aldrich (St. Louis, MO, USA). PHA543613 hydrochloride (PHA) was obtained from Tocris Bioscience (Ellisville, MO, USA). Dulbecco’s Modified Eagle’s Medium (DMEM), fetal bovine serum (FBS), horse serum (HS) were from Hyclone Laboratories (Logan, UT). HALT^TM^ protease inhibitor cocktail and bicinchoninic acid (BCA) reagent kit were from Pierce (Rockford, IL, USA). Anti-phospho-PKB (Ser-473), anti-phospho-MAPK, and horseradish-peroxidase (HRP) conjugated goat anti-rabbit IgG secondary antibodies were from Cell Signaling Technology (Danvers, MA, USA). ECL Advance Blocking Reagent and Western Lightning *plus*-ECL Enhanced Chemiluminescent Substrate were from GE Healthcare Biosciences (Piscataway, NJ, USA). 

Highly purified saxitoxin dihydrochloride (**STX-diHCl**) quantified by NMR was provided by the NRCC Certified Reference Material program (Halifax, NS, Canada). Deionized ultrapure water from a Milli-Q system from Millipore (Bedford, MA, USA) was used in all preparations and manipulations requiring aqueous solutions. 

### 2.1. Algal Material

The *A. ostenfeldii* cultures AOSH1 and AOSH2 were initiated from single cell isolates from plankton samples collected at Ship Harbour, Nova Scotia, Canada in 2000, whereas HT140E7 was obtained from a single cell isolate from plankton samples collected in the Gulf of Maine, USA. The clonal isolates were identified by Nomarski contrast interference microscopy, and confirmed by epifluorescence microscopy after staining the thecal plates with calcofluor [13]. The cultures are maintained at the National Research Council’s Institute for Marine Biosciences. 

### 2.2. Culturing of *A. ostenfeldii* Clonal Isolates

AOSH1, AOSH2 and HT140E7 cultures were initiated in L1 growth medium diluted 1:10 with sterile seawater in multiwell tissue culture plates. Cultures were scaled-up in full-strength L1 medium by serial transfer into 250 mL Erlenmeyer flasks and then into 2.8 L Fernbach flasks, to a minimum total culture volume of 80 L. Unialgal cultures were maintained at an ambient photon flux density of 90 mmol m^−2^ s^−1^ at 14 °C and a 14:10 h light/dark photocycle in a controlled growth chamber. Cells were harvested in late exponential growth phase by gravity filtration onto a 20 mm Nitex mesh sieve and concentrated by centrifugation (2630 g) for 15 min.

### 2.3. Isolation of Spirolides from Cultured *A. ostenfeldii*

Isolation of spirolide **13dmC** was isolated from AOSH1, 20-methyl spirolide G (**20mG**), spirolide C (**C**) and spirolide H (**H**) from AOSH2 and spirolides A (**A**) and B (**B**) from HT140E7 using previously reported spirolide isolation schemes [14,15,16]. Briefly, thawed wet cell pellets of AOSH1, AOSH2 and HT140E7 were extracted four times using sonication in the presence of methanol. After centrifugation, the methanolic supernatants were pooled and evaporated to dryness. The residue was dissolved in water and partitioned with dichloromethane. The resulting dichloromethane extract was dissolved in methanol and applied to an LH-20 column. Spirolide-containing fractions were dissolved in 30% methanol and subjected to a C_18_ flash chromatography column, which was conditioned and eluted with 40% acetonitrile (0.1% trifluoroacetic acid). Fractions containing spirolides were combined and evaporated to dryness. Purification of **13dmC**, **20mG** and **C** containing fractions was accomplished using a Vydac 201TP510 C_18_ HPLC column (Mandel Scientific Company Ltd., Ontario, Canada), which was eluted isocratically with 30% acetonitrile (0.1% trifluoroacetic acid) and monitored at 210 nm. Fractions containing **A** and **B** were purified using a BDS Hypersil C_8_ HPLC column, which was eluted isocratically with 30% acetonitrile (0.1% trifluoroacetic acid) and monitored at 210 nm. The identity and high purity of the isolated spirolides was confirmed by NMR and quantified using quantitative NMR before being subjected to toxicity evaluation.

### 2.4. Instrumentation

An Agilent 1100 Series HPLC (Palo Alto, California, USA) with a binary pump system equipped with a diode array detector (DAD) and ChemStation^®^ software was used for the final stage of purification of the spirolides used in this study. All NMR measurements were collected on a Bruker DRX-500 spectrometer (Bruker, Canada) at 500.13 MHz (1H) and 125.77 MHz (^13^C). Samples were dissolved in 0.5 mL of CD_3_OH and run at 20 °C. Spectra were referred to CHD_2_OH at 3.3 ppm (^1^H) or ^13^CD_3_-OH at 49.0 ppm (^13^C). Standard Bruker pulse sequences were used with solvent suppression by presaturation where appropriate. Structural assignments for each spirolide studied were confirmed via comparison to NMR data available in the literature.

### 2.5. Cell Culture

*Cytotoxicity*. The potential cytotoxic effect of **13dmC** was tested in several cell culture lines obtained from the American Type Culture Collection (Manassas, VA). These were HepG2 (HB-8065; liver), NIE-115 (CRL-2263; neuroblastoma), 3T3-L1 (CL-173; adipocyte), SKOV-3 (HTB-77; ovarian cancer), C2C12 (CRL-1772; skeletal muscle), and RAW264.7 (TIB-71; macrophage) cells. Cells were grown according to instructions from the culture collection. They were seeded at a density of 5000 cells per well in 96-well plates and grown for 48-h before use. Cells were treated with **13dmC** (100 nM) for approximately 24-h and then examined for evidence of cytotoxicity by monitoring lactate dehydrogenase (LDH) release (CytoTox-ONE™; Promega Corporation), mitochondrial respiration (MTS assay, CellTiter 96^®^; Promega Corporation, Madison, WI), and lysosomal function (neutral red (NR) assay). 

*Nicotinic AChR signaling.* C2C12 mouse muscle cells were seeded in 12-well plated and grown to DMEM containing 2 mM L-glutamine plus 10% fetal bovine serum. They were induced to differentiation into myotubes, by switching to DMEM containing 2 mM L-glutamine plus 2% horse serum (HS). The characterization of cells as myotubes was based on visual observation of multinucleated cells as well as spontaneous contraction. All cell treatments were conducted in DMEM plus 0.2% HS at 37 °C in a cell culture incubator (5% CO_2_). Experiments were initiated by incubating cells in this medium for 1–2 h after which time they were treated with **13dmC** (100 nM) or vehicle (methanol + 0.1% TFA) for 30 min. Cells were then treated for 20 min with either 10 μM nicotine, 10 μM PHA, or 100 nM insulin. Control wells received the appropriate amount of vehicle (1 μL/mL). At the end of the treatment period cells were rinsed once with ice-cold phosphate buffered saline and lysed on ice with lysis buffer (50 mM Tris, pH 8.0, 150 mM NaCl, 10 mM NaF, 10 mM β-glycerophosphate, 10 mM Na pyrophosphate, 5 mM sodium vanadate, 1% Triton X-100, 0.1% SDS, and 1% HALT^TM^ protease inhibitor cocktail). Cell lysate protein concentration was determined using the BCA method with bovine serum albumin as standard. Lysates were subjected to SDS-PAGE and transferred onto PVDF membrane. Membranes were blocked for 1 h at room temperature with blocking solution (TBS, 0.1% tween, 5% skim milk powder), and incubated with anti-phospho-PKB (Ser-473) and anti-phospho-MAPK antibodies overnight with shaking at 4 °C. After washing with wash solution (TBS, 0.1% tween), the membranes were treated horseradish-peroxidase conjugated goat anti-rabbit IgG secondary antibody in 5% ECL Advance Blocking Reagent for 1 h at room temperature. Washed membranes were then developed using ECL Western Lightning *plus*-ECL Enhanced Chemiluminescent Substrate. 

### 2.6. Determination of Acute Toxicities

Acute toxicities were determined according to the principles of OECD Guideline 425 [17]. LD_50_ values (median lethal doses) and their 95% confidence limits were calculated using the AOT 425 Statistical Program [18]. Each determination of the LD_50_ and 95% confidence interval required between 6 and 9 animals. Swiss albino mice were bred at the Ruakura Research Centre, Hamilton, New Zealand. Female mice, 10 weeks of age, weighing between 18 and 22 g, were used in all experiments. They were housed in solid-bottomed cages containing bedding of softwood shavings. In most experiments, mouse food (Laboratory Chow, Sharpes Animal Feeds, Carterton, New Zealand) was available *ad libitum* (“fed mice”). In some experiments, however, mice were deprived of food from 4 p.m. until dosing at 8–9 a.m. the next morning (“fasted mice”). Food was returned to these mice immediately after dosing. All animals were allowed access to water at all times. 

The test materials were dissolved in 95% ethanol. Mice were weighed immediately before dosing and test materials were administered on a µg/kg body weight (BW) basis. Mice were monitored intensively during the day of dosing and those dying during the course of the experiment were subjected to macroscopic examination, while survivors were examined and weighed each day for 14 days after administration of the test compounds. They were then killed and necropsied.

For intraperitoneal (ip) injection, the ethanolic solution was diluted with 1% Tween-60 in normal saline and 95% ethanol added as necessary to maintain a uniform concentration of 5% (v/v) in the solution. The total volume administered was 1 mL. For gavage, aliquots of the spirolide solutions were diluted with Tween-saline to give a total volume of 200 µL, containing 12.5% (v/v) ethanol. For determination of toxicity by feeding, aliquots of the spirolide solutions were absorbed onto dry mousefood (~150 mg) or mixed with moist powdered mousefood (~150 mg) and given to fasted mice. Alternatively, the solutions were mixed with Tararua Traditional Cream Cheese (~150 mg; Goodman Fielder New Zealand Ltd, Auckland, NZ). Some mice were reluctant to eat dry mousefood containing spirolide derivatives and only those animals consuming all the food in less than 1 min were used for calculating LD_50_ values. All mice given spirolides mixed with moist mousefood or with cream cheese ate all the food within 1 min.

## 3. Results

### 3.1. Effects of Spirolides on Cells in Culture

**Figure 1:**
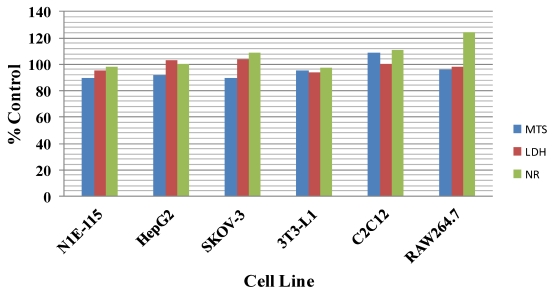
Effect of 13-desmethyl C spirolide on the viability of various cell types grown in culture. Cells were incubated for 24 h in the presence of 100 nM 13-desmethyl C spirolide. Control cells were incubated with vehicle in the absence of toxin. Results are the average of two experiments performed in duplicate. Following incubation cytotoxicity was assessed using measures of mitochondrial respiration (MTS assay), plasma membrane integrity (LDH release), and lysosomal function (NR staining). Results were normalized to those of control wells.

When cell lines, representative of various tissue types, were treated with 100 nM **13dmC** for 24-h, no evidence of cytotoxicity was revealed using 3 different assay methods to monitor cell integrity and function (Figure 1). Similar results were observed when cells were treated with **20mG** or **H** (results not shown). Higher concentrations of spirolide were not tested because of the limited amounts of the test materials that were available.

#### Effect of **13dmC** on Cell Signaling from the Nicotinic AChR

**Figure 2:**
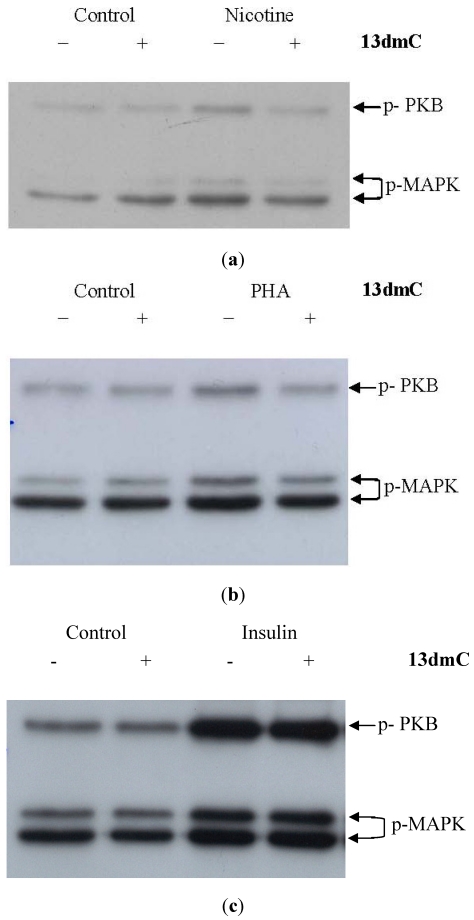
Phosphorylation of PKB and MAPK by nicotinic agonists is inhibited by **13dmC**. Fully differentiated C2C12 myotubes were pre-treated with **13dmC** (100 nM; “+”) or vehicle (methanol + 0.1% trifluoracetic acid; “−”) for 30 min. Cells were then treated with: nicotine (10 μM; panel **a**), PHA543613 (PHA, 10 µM; panel **b**), or insulin (100 nM; panel **c**) for 20 min. Corresponding controls received vehicle alone. Cell lysates were analyzed by immunoblot for p-473 PKB (MW 60 kDa) and p-MAPK (MW 42 and 44 kDa) using phosphospecific antibodies. These are representative blots from 2–3 independent experiments.

The expression of the α-7 subtype of the nAChR increases during differentiation of C2C2 (results not shown). Stimulation of fully differentiated C2C12 cells with nicotine leads to phosphorylation (activation) of downstream signaling molecules including MAPK and PKB (Figure 2a). However, when cells were pre-treated with **13dmC** this stimulation of MAPK and PKB was diminished (Figure 2a). The same inhibitory effects of **13dmC** were also observed when cells were stimulated with PHA, an α7 nAChR agonist (Figure 2b). In contrast, the stimulation of PKB and MAPK by insulin was unaffected by **13dmC** pre-treatment of the cells (Figure 2c). 

### 3.2. Acute Toxicities

The median lethal doses of the spirolide derivatives by ip injection are shown in Table 1. By this route, **C**, **13dmC** and **20mG** were highly toxic, while **A** and **B** were significantly less so. The state of alimentation of the mice (fed *versus* fasted) had no effect on the acute ip toxicity of **13dmC**. The spirolide derivatives were also toxic to mice after oral administration via gavage, although the median lethal doses by this route were significantly lower than those by ip injection—in fed mice, the LD_50_ values were between 15 and 23 times higher. By gavage, however, there was a marked effect of state of alimentation, with fasted mice being significantly more susceptible, by factors of between 1.3 and 3.3 (Table 2). 

**Table 1:** Acute toxicity of spirolides by ip injection in mice.

Compound	State of alimentation	LD_50_ (µg/kg body weight) *^a^*	LD_50_ (µmol/kg body weight) *^a^*
Spirolide A	Fed	37 (35–44)	0.054 (0.051–0.064)
Spirolide B	Fed	99 (ND)	0.14 (ND)
Spirolide C	Fed	8.0 (4.6–16)	0.011 (0.0065–0.023)
13-Desmethyl spirolide C	Fed	6.9 (5.0–8.0)	0.010 (0.0072–0.012)
13-Desmethyl spirolide C	Fasted	6.9 (5.0–8.0)	0.010 (0.0072–0.012)
20-Methyl spirolide G	Fed	8.0 (3.9–14)	0.011 (0.0055–0.020)

*^a^* Figures in brackets indicate 95% confidence intervals. ND: Not determined. In these cases, the pattern of deaths were such that the AOT program was unable to calculate confidence intervals.

**Table 2:** Acute toxicity of spirolides by gavage in mice.

Compound	State of alimentation	LD_50_ (µg/kg body weight) *^a^*	LD_50_ (µmol/kg body weight) *^a^*
Spirolide A	Fed	550 (436–690)	0.80 (0.63–1.0)
Spirolide C	Fed	180 (ND)	0.25 (ND)
13-Desmethyl spirolide C	Fed	160 (123–198)	0.23 (0.18–0.29)
20-Methyl spirolide G	Fed	160 (ND)	0.23 (ND)
Spirolide A	Fasted	240 (188–298)	0.34 (0.27–0.43)
Spirolide B	Fasted	440 (320–500)	0.63 (0.46–0.72)
Spirolide C	Fasted	53 (50–63)	0.075 (0.071–0.089)
13-Desmethyl spirolide C	Fasted	130 (87–166)	0.18 (0.13–0.24)
20-Methyl spirolide G	Fasted	88 (27–120)	0.13 (0.038–0.17)

*^a^* Figures in brackets indicate 95% confidence intervals. ND: Not determined. In these cases, the pattern of deaths were such that the AOT program was unable to calculate confidence intervals.

The toxicities of the spirolides were even lower when administered by feeding (Table 3). In comparative studies with **13dmC**, it was found that some mice were reluctant to eat dry mousefood to which this substance had been added, although enough mice rapidly ate the feed to permit estimation of the LD_50_. In contrast, mice readily ate moist mousefood or cream cheese contaminated with **13dmC**. The vehicle employed for administration (dry mousefood, moist mousefood or cream cheese) had no significant effect on the acute toxicity of this substance. Again, fasted mice were more sensitive than fed animals (Table 3).

**Table 3:** Acute toxicity of spirolides by feeding to mice.

Compound	State of alimentation	Vehicle	LD_50_ (µg/kg body weight) *^a^*	LD_50_ (µmol/kg body weight) *^a^*
Spirolide A	Fed	Cream cheese	1300 (1250–1580)	1.9 (1.8–2.3)
Spirolide C	Fed	Cream cheese	780 (ND)	1.1 (ND)
13-Desmethyl spirolide C	Fed	Cream cheese	1000 (861–1290)	1.5 (1.2–1.9)
20-Methyl spirolide G	Fed	Cream cheese	630 (476–882)	0.89 (0.68–1.3)
Spirolide A	Fasted	Cream cheese	1200 (1047–3690)	1.7 (1.5–5.4)
Spirolide C	Fasted	Cream cheese	500 (353–657)	0.71 (0.50–0.93)
13-Desmethyl spirolide C	Fasted	Dry mousefood	630 (547–829)	0.90 (0.79–1.2)
13-Desmethyl spirolide C	Fasted	Moist mousefood	590 (500–625)	0.85 (0.72–0.90)
13-Desmethyl spirolide C	Fasted	Cream cheese	500 (381–707)	0.72 (0.55–1.0)
20-Methyl spirolide G	Fasted	Cream cheese	500 (381–707)	0.71 (0.54–1.0)

*^a^* Figures in brackets indicate 95% confidence intervals. ND: Not determined. In these cases, the pattern of deaths were such that the AOT program was unable to calculate confidence intervals.

The symptoms of intoxication were the same for all the spirolide derivatives and for all routes of administration. At lethal doses, the animals became hyperactive immediately after dosing, moving with a rather unsteady gait. Abdominal breathing was noted soon after, and the mice moved with a rolling gait, their hind legs being partially extended. Subsequently, the animals became prostrate, with their hind legs fully extended. Their respiration rates progressively declined, with occasional gasps, until respiration ceased completely. Rapid flicking movements of the hind legs occurred immediately before death, together with severe exophthalmia. After injection or gavage, death occurred between 3 and 20 min after administration of the test substances. The onset of symptoms was delayed in mice given spirolides by feeding, and deaths occurred at up to 35 min after dosing. At toxic, but sublethal doses, the spirolides induced immobility, accompanied by rapid, shallow, abdominal breathing and extension of the hind legs. Recovery was rapid, however, and within an hour after dosing the appearance and behavior of the mice became normal, and remained so throughout the subsequent 14-day observation period. No macroscopic abnormalities were recorded in any of the animals at necropsy.

## 4. Conclusions

By ip injection, all the spirolide derivatives tested were toxic (Table 1). The median lethal doses of **C**, **13dmC** and **20mG** were similar, at 6.9–8.0 µg/kg (0.010–0.011 µmol/kg), while those of **A** and **B** were respectively ~5 and 13 times lower. The relatively low acute toxicity of **B** is consistent with an earlier report of an LD_100_ of 250 µg/kg [1]. The symptoms of intoxication were the same with all the derivatives, and were very similar to those recorded with gymnodimine, a related cyclic imine [19], with death resulting from respiratory failure. Like gymnodimine, the spirolides appeared to be rapidly absorbed, since symptoms of intoxication were seen soon after administration. Mice given doses of the spirolides that were not lethal but were sufficient to cause marked toxicity recovered rapidly with no perceptible long-term effects, suggesting that these compounds are rapidly detoxified and/or excreted. 

Addition of spirolides to a variety of cell lines did not produce obvious cytotoxicity, as indicated by various indicators of viability. Our results are consistent with recent findings showing lack of toxicity in the BE(2)-M17 neuroblastoma cell line exposed to 10 nM, 100 nM and 500 nM **13dmC** for periods up to 24 h [12]. In BE(2)-M17 cells 100 nM **13dmC** effectively blocked the acetylcholine-induced calcium signal and reduced binding of a specific muscarinic receptor agonist to the plasma membrane, suggesting interaction with muscarinic-type AChRs [12]. In keeping with reports that spirolides also inhibit nicotinic type AChRs [11], we found that 100 nM **13dmC** inhibited the stimulation of C2C12 by nAChR agonists. We observed an inhibition of MAPK and PKB, enzymes which become phosphorylated in response to nAChR stimulation. The observation that **13dmC** did not inhibit insulin stimulation of MAPK and PKB is consistent with the AChR target for these toxins. Thus while not cytotoxic to a variety of cell lines *per se*, the presence of **13dmC** affects receptor function with influences on various cellular processes.

Like many other toxins, such as gymnodimine [19], yessotoxin [20], saxitoxin [21], and palytoxin [22], the spirolides were less toxic by gavage than by ip injection (Table 2). By this route of administration, however, there was a significant effect of the state of alimentation, with fasted mice being more susceptible than fed. Fasting has been shown to increase the acute toxicity of many compounds [23,24,25,26], either through inhibition of detoxification processes [25,27,28] or through an increase in the rate of transfer of the test material from the stomach to the small intestine, thus facilitating absorption [24]. In the case of the spirolides, the former possibility is unlikely. Effects on detoxification would be expected to influence toxicity by any route of administration, but it was found that fasting had absolutely no effect on the acute toxicity of **13dmC** by injection. In both fed and fasted mice, **C**, **13dmC** and **20mG** were more toxic than **A**. It is therefore likely that an increase in the rate of passage of the toxin from the stomach to the small intestine is responsible for the higher toxicity observed in fasted mice. Spirolide **B** was of comparatively low toxicity in fasted mice; insufficient material was available to determine the acute toxicity of this substance by gavage in fed mice.

In order to express systemic toxicity, toxins must be absorbed, and the rate of transfer of toxins into the small intestine is therefore crucially important for determining the severity of toxicity. This will be influenced not only by fasting but also by the method of administration. In mice, because of the semi-solid consistency of the stomach contents, test materials may flow around the mass of undigested food and rapidly enter the duodenum at high concentration. In contrast, material ingested by humans immediately becomes mixed and diluted with the liquid contents of the stomach, and the diluted material is then gradually released into the duodenum. It has been argued that administration of toxins to mice by gavage may give an artefactually high estimate of the risk of such compounds to human health [29], and that administration by feeding may be a more relevant method of dosing toxins to mice [19,22]. By feeding, the test material becomes uniformly distributed through the stomach contents of the mouse, and is gradually released into the small intestine, thus more closely reproducing the situation in the human.

In order to estimate the acute toxicity of a compound by feeding, it is essential that the toxin-contaminated food is rapidly eaten by the animal. Fasted mice will readily eat small quantities of mousefood, while mice, whether fed or fasted, will immediately eat cream cheese. In the present experiments, some fasted mice appeared to be able to detect the presence of **13dmC** in dry mousefood, since they were unwilling to consume the contaminated food. When the test substance was more uniformly distributed through moist mousefood, however, it was readily accepted by the animals. The LD_50_ found via this method of administration was very similar to that using dry mousefood. The LD_50_ determined by using cream cheese as the vehicle was not significantly different from those found using mousefood, indicating that the high fat content of the cheese (33%) did not delay the uptake of the test material or decrease the rate of gastric emptying. For establishing the acute toxicities of the other spirolides by feeding, only cream cheese was used, since with this vehicle toxicity to both fed and fasted mice could be determined. 

Insufficient **B** was available to determine acute toxicities by feeding. By this route, **A** was again less toxic than **C**, **13dmC** and **20mG** (Table 3). In fasted mice, the LD_50_s of the last-named three compounds were very similar (500–625 µg/kg (0.71–0.90 µmol/kg)), but more variation (630–1000 µg/kg (0.89–1.5 µmol/kg)) was observed in fed mice, possibly reflecting differences in the amount of food in the animals’ stomachs. Indeed, the primary reason for the use of prolonged (16–24 h) fasting in acute toxicity studies is to decrease variability in response, though it has been suggested, in view of the significant effects on the acute toxicity of many chemicals, and the lack of relevance to the human situation, that such long-term fasting should be avoided [24,26]. Furthermore, since seafood is generally consumed as part of a meal, fed animals would appear to be the more appropriate model for the evaluation of toxins present in this foodstuff.

When the five spirolides were tested for acute toxicity in mice by ip injection, by gavage or by feeding, the same trend in toxic response was observed in which **C**, **13dmC** and **20mG** were more toxic than **A** and **B**. This observation suggests that the cyclic imine ring C-31 methyl group which is present in **C**, **13dmC** and **20mG**, but not **A** and **B**, could have a significant influence on the toxic response.

In the cyclic imine family of algal toxins, of which the spirolides are members, the imine moiety is a necessary condition for toxicity [30]. When the imine group is reduced, forming dihydrospirolide B, or destroyed by ring-opening, as in spirolides E and F, the toxicity is greatly decreased [9]. It is not, however, a sufficient condition for expression of toxic effects, since **H**, which, although containing the imine function, was of low toxicity to mice by ip injection, with only transient effects being observed at a dose of 2,000 µg/kg [16]. 

In order to compare the spirolide potencies with a currently regulated toxin, identical toxicity testing was performed on **STX-diHCl**, the principal toxin responsible for paralytic shellfish poisoning (PSP). The LD_50_ values determined for **STX-diHCl** were as follows: ip (fed)–0.027 (0.018–0.040) μmol/kg BW; gavage (fed)–4.3 (3.6–4.5) μmol/kg BW; and feeding (fed, via cream cheese)–14 (6.9–25) μmol/kg BW. It can be observed from this data that spirolides are more toxic orally on a molar basis than **STX-diHCl** which is a regulated PSP toxin.

In this study the ip LD_50_ values of **13dmC** and 20mG were determined to be 6.9 µg/kg BW (95% confidence interval of 5.0–8.0 µg/kg BW) and 8.0 µg/kg BW (95% confidence interval of 3.9–14.0 µg/kg BW) respectively. In a recent publication, the LD_50_ of **13dmC** was reported as 27.9 µg/kg BW, while for 20mG no deaths were reported at doses up to 63.5 µg/kg BW [31]. Our investigations therefore showed higher levels of toxicity for these two spirolides. Inter-laboratory variations such as these have been previously reported for toxicity estimations; an example being with yessotoxin in which LD_50_ values of 80 to 512 µg/kg BW have been reported in the literature [32]. Such differences may be attributable to the purity of the test compound, or differences in animal strains or sex used in the studies. Our studies utilized female mice and high purity toxins which were accurately quantitated using quantitative NMR. The sex of the animals in the recent study was not specified.

Spirolides are not currently regulated and at present there is no conclusive evidence that contamination of seafood with these substances has caused human intoxication. Episodes of toxicity, involving rather non-specific symptoms such as gastric distress and tachycardia, were recorded in individuals in Nova Scotia consuming shellfish during times when spirolides were known to be present, but these could not definitively be ascribed to spirolides [10]. As shown in the present work, however, spirolides are toxic not only by injection but also after oral administration, and with the widespread distribution of spirolide-producing strains of *A. ostenfeldii*, the possible impact of these substances on human health must be considered. The present study will provide toxicity equivalence factors if spirolides are regulated in the future, when detailed short-term feeding studies will be required in order to establish guidance levels for these compounds.

## References

[1] Hu T., Curtis J.M., Oshima Y., Quilliam M.A., Walter J.A., Watson-Wright W.M., Wright J.L.C. (1995). Spirolides B and D, two novel macrocycles isolated from the digestive glands of shellfish. J. Chem. Soc. Chem. Commun..

[2] Uemura D., Chou T., Haino T., Nagatsu A., Fukuzawa S., Zheng S., Chen H. (1995). Pinnatoxin A: A toxic amphoteric macrocycle from the Okinawan bivalve *Pinna muricata*. J. Am. Chem. Soc..

[3] Takada N., Umemura N., Suenaga K., Chou T., Nagatsu S., Haino T., Yamada K., Uemura D. (2001). Pinnatoxins B and C, the most toxic components in the pinnatoxins series from the Okinawan bivalve *Pinna muricata*. Tetrahedron Lett..

[4] Lu C.-K., Lee G.-H., Huang R., Chou H.-N. (2000). Spiro-prorocentrimine, a novel macrocyclic lactone from a benthic *Prorocentrum* sp. of Taiwan. Tetrahedron Lett..

[5] Seki T., Satake M., MacKenzie L., Kasper H.F., Yasumoto T. (1995). Gymnodimine, a new marine toxin of unprecedented structure isolated from New Zealand oysters and the dinoflagellate, *Gymnodinium* sp.. Tetrahedron Lett..

[6] Cembella A.D., Lewis N.I., Quilliam M.A. (1999). Spirolide composition in micro-extracted pooled cells isolated from natural plankton assemblages and from cultures of the dinoflagellate *Alexandrium ostenfeldii*. Nat. Toxins.

[7] Cembella A.D., Lewis N.I., Quilliam M.A. (2000). The marine dinoflagellate *Alexandrium ostenfeldii* (Dinophyceae) as the causative organism of spirolide shellfish toxins. Phycologica.

[8] Hansen P.J., Cembella A.D., Moestrup O. (1992). The marine dinoflagellate *Alexandrium ostenfeldii*: Paralytic shellfish toxin concentration, composition, and toxicity to a tintinnid ciliate. J. Phycol..

[9] Hu T., Curtis J.M., Walter J.A., Wright J.L.C. (1996). Characterization of biologically inactive spirolides E and F: Identification of the spirolide pharmacophore. Tetrahedron Lett..

[10] Richard D., Arsenault E., Cembella A., Quilliam M., Hallegraeff G.M., Blackburn S.I., Bolch C.J., Lewis R.J. Investigations into the Toxicology and Pharmacology of Spirolides, a Novel Group of Shellfish Toxins. Proceedings of the 9th International Conference on Harmful Microalgae.

[11] Bourne Y., Radic Z., Araoz R., Talley T.T., Benoit E., Servent D., Taylor P., Molgo J., Marchot P. (2010). Structural determinants in phycotoxins and AChBP conferring high affinity binding and nicotinic AChR antagonism. Proc. Natl. Acad. Sci. USA.

[12] Wandscheer C.B., Vilarino N., Espina B., Louzao M.C., Botana L.M. (2010). Human muscarinic acetylcholine receptors are a target of the marine toxin 13-desmethyl C spirolide. Chem. Res. Toxicol..

[13] Fritz L., Triemer R.E., Anderson D.M., White A.W., Baden D.G. (1985). Preliminary Studies of Cell Wall Formation in Temporary Cysts of *Gonyaulax tamarensis*. Toxic Dinoflagellates.

[14] Hu T., Burton I.W., Cembella A.D., Curtis J.M., Quilliam M.A., Walter J.A., Wright J.L.C. (2001). Characterization of spirolides A, C and 13-desmethyl C, New marine toxins isolated from toxic plankton and contaminated shellfish. J. Nat. Prod..

[15] Aasen J., MacKinnon S.L., LeBlanc P., Walter W.A., Hovgaard P., Aune T., Quilliam M.A. (2005). Detection and identification of spirolides in Norwegian shellfish and plankton. Chem. Res. Toxicol..

[16] Roach J.S., LeBlanc P., Lewis N.I., Munday R., Quilliam M.A., MacKinnon S.L. (2009). Characterization of a dispiroketal spirolide subclass from *Alexandrium ostenfeldii*. J. Nat. Prod..

[17] OECD (2001). OECD guideline for testing of chemicals 425. Acute oral toxicity-up and down procedure. http://www.epa.gov/oppfead1/harmonization/docs/E425guideline.pdf.

[18] EPA User documentation for the AOT425StatPgm program prepared for the US Environmental Protection Agency by Westat, May 2001; updated by USEPA September 2002. http://www.oecd.org/dataoecd/19/57/1839830.pdf.

[19] Munday R., Towers N.R., Mackenzie L., Beuzenberg V., Holland P.T., Miles C.O. (2004). Acute toxicity of gymnodimine to mice. Toxicon.

[20] Aune T., Sørby R., Yasumoto T., Ramstad H., Landsverk T. (2002). Comparison of oral and intraperitoneal toxicity of yessotoxin towards mice. Toxicon.

[21] Harada T., Oshima Y., Yasumoto T. (1984). Assessment of potential activation of gonyautoxin V in the stomach of mice and rats. Toxicon.

[22] Munday R. (2006). Toxicological requirements for risk assessment of shellfish contaminants: A review. Afr. J. Mar. Sci..

[23] Dashiell O.L., Kennedy G.L. (1984). The effects of fasting on the acute oral toxicity of nine chemicals in the rat. J. Appl. Toxicol..

[24] Kast A., Nishikawa J. (1981). The effect of fasting on oral acute toxicity of drugs in rats and mice. Lab. Anim..

[25] Massey R.M., McElligott T.E., Racz W.J. (1982). Acetaminophen toxicity in fed and fasted mice. Can. J. Physiol. Pharmacol..

[26] Strubelt O., Dost-Kempf E., Siegers C.-P., Younes M., Völpel M., Preuss U., Dreckmann J.G. (1981). The influence of fasting on the susceptibility of mice to hepatotoxic injury. Toxicol. Appl. Pharmacol..

[27] Chavanet P., Joly V., Rigaud D., Bolard J., Carbon C., Yeni P. (1994). Influence of diet on experimental toxicity of amphotericin B deoxycholate. Antimicrob. Agents Chemother..

[28] Price V.F., Miller M.G., Jollow D.J. (1987). Mechanisms of fasting-induced potentiation of acetaminophen hepatotoxicity in the rat. Biochem. Pharmacol..

[29] Mantle P.G. (2002). Risk assessment and the importance of ochratoxins. Int. Biodeterior. Biodegrad..

[30] Munday R., Botana L.M. (2008). Toxicology of Cyclic Imines: Gymnodimine, Spirolides, Pinnatoxins, Pteriatoxins, Prorocentrolide, Spiro-Procentrimine and Symbioimines. Seafood and Freshwater Toxins, Pharmacology, Physiology and Detection.

[31] Otero P., Alfonso A., Rodriguez P., Rubiolo J.A., Cifuentes J.M., Bermudez R., Vieytes M.R., Botana L.M.  (2011). Pharmacokinetic and toxicological data of spirolides after oral and intraperitoneal administration. Food Chem. Toxicol..

[32] Munday R., Aune T., Rossini G.P., Botana L.M. (2008). Toxicology of the Yessotoxins. Seafood and Freshwater Toxins, Pharmacology, Physiology and Detection.

